# COVID-19 vaccine-associated ganulomatous mass mimicking a sarcoma: a case report^☆^

**DOI:** 10.1016/j.radcr.2022.05.035

**Published:** 2022-06-03

**Authors:** Daniel Quintero, Nikhil Patel, Griffin Harris, Anthony Maristany, Ali Alani, Andrew E Rosenberg, Sheila A Conway, Jean Jose

**Affiliations:** a University of Miami Leonard Miller School of Medicine, Miami, FL, USA; b University of Miami, Leonard Miller School of Medicine, Department of Orthopedic Surgery, Miami, FL, USA; c Florida International University Herbert Wertheim College of Medicine, Miami, FL, USA; d University of Miami, Leonard Miller School of Medicine, Department of Pathology, Miami, FL, USA; e University of Miami, Leonard Miller School of Medicine, Department of Radiology, Miami, FL, USA

**Keywords:** Benign COVID-19 complications, Musculoskeletal radiology, Soft tissue tumor

## Abstract

Several studies have previously documented the development of complications stemming from injection with one of the various COVID-19 vaccines. No study, however, has discussed the spontaneous development of a soft tissue mass shortly after a COVID-19 vaccine injection. We report on 66-year-old female with concerns of a growing shoulder mass, 2 weeks after receiving a COVID-19 vaccine booster. Initial work-up with X-ray and MRI was concerning for a soft tissue neoplasm, specifically a soft tissue sarcoma. Subsequent ultrasound guided biopsy demonstrated a benign granulomatous lesion. No further management was required as the lesion spontaneously resolved during a 3-month follow-up period.

## Introduction

It has been previously documented that vaccine components can produce granulomatous inflammation. One study reported postvaccination granulomas in children following the pertussis immunization [1]. This same group identified that the ingredient responsible was an aluminum salt adjuvant as 77% of children who developed granulomatous inflammation also developed a contact allergy to aluminum [1]. The etiology of this granulomatous reaction was determined to be a delayed hypersensitivity reaction. The COVID-19 Vaccine has been previously associated with anaphylaxis, thrombosis, and thrombocytopenia. To date, there have been no reports on the formation of soft tissue mass, secondary to a COVID-19 vaccination. We present the first case of a soft tissue growth with malignant characteristics that developed shortly after vaccination. We propose that COVID-19 vaccine similarly manifests a granulomatous reaction by inducing a delayed hypersensitivity reaction.

## Case report

A 66-year-old female presents to our orthopedic clinic with concerns of a right shoulder rapidly expanding palpable mass. This female with a medical history of rheumatoid arthritis (RA) and rotator cuff surgery presents to our outpatient orthopedic sports medicine clinic concerned about a mildly tender and rapidly enlarging right shoulder soft tissue mass of three weeks duration. She denies systemic symptoms of fever, appetite changes, or weight loss, as well as any antecedent major trauma or other injury. The patient had received the COVID-19 Moderna vaccine booster (3rd dose) in the same deltoid region 4 weeks prior. She notated the injection had been associated with more pain than the prior doses.

**Fig. 1:**
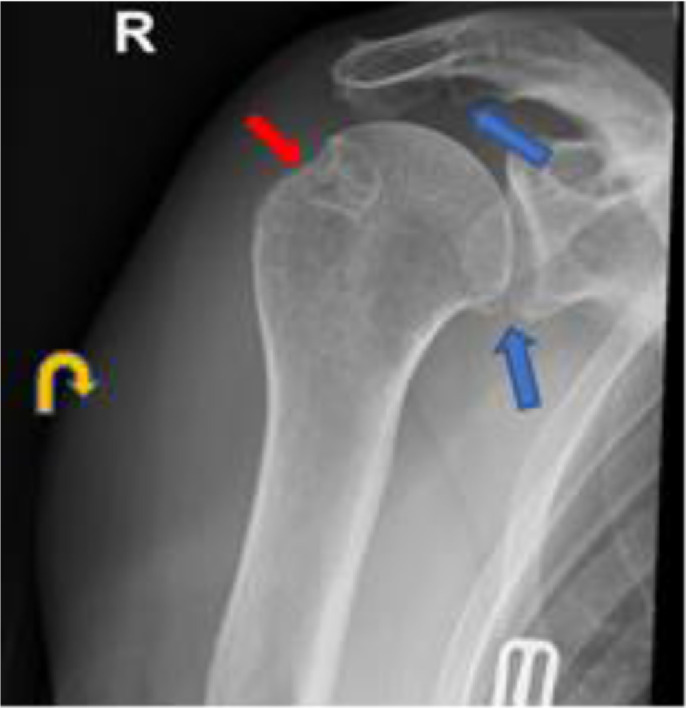
Frontal radiograph of the right shoulder demonstrates osteoarthritis of the acromioclavicular and glenohumeral joints with small marginal osteophytes (blue arrows). There is subacromial spurring and post-surgical changes of the greater tuberosity following prior rotator cuff repair (red arrows). There is a noncalcified soft tissue mass along the lateral aspect of the shoulder (curved yellow arrows), without evidence of underlying periosteal reaction, erosion, lytic or blastic osseous lesion.

**Fig. 2:**
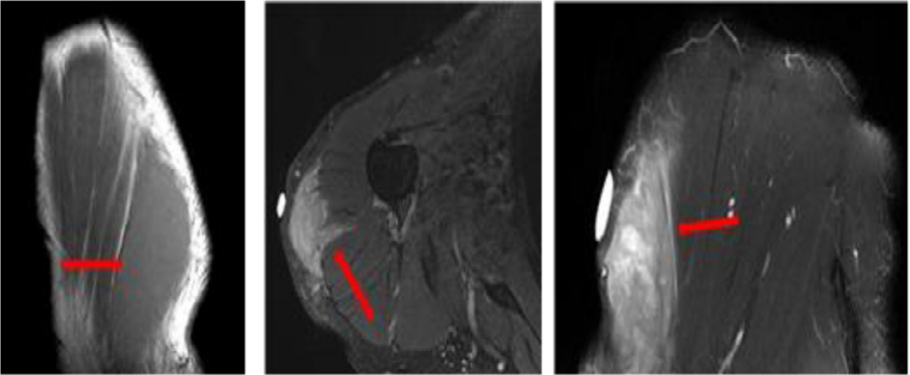
(A) Sagittal T1, (B) axial PDFS, and (C) Coronal T2FS MRI of the right shoulder demonstrate a solid heterogenous soft tissue mass involving the lateral belly of the deltoid muscle and extending laterally into the subcutaneous fat, over the area of palpable abnormality as demarcated by the external marker (red arrows). The lesion was mildly hyperintense to skeletal muscle on T1-weighted images, and more pronouncedly hyperintense to skeletal muscle on T2-weighted images.

**Fig. 3:**
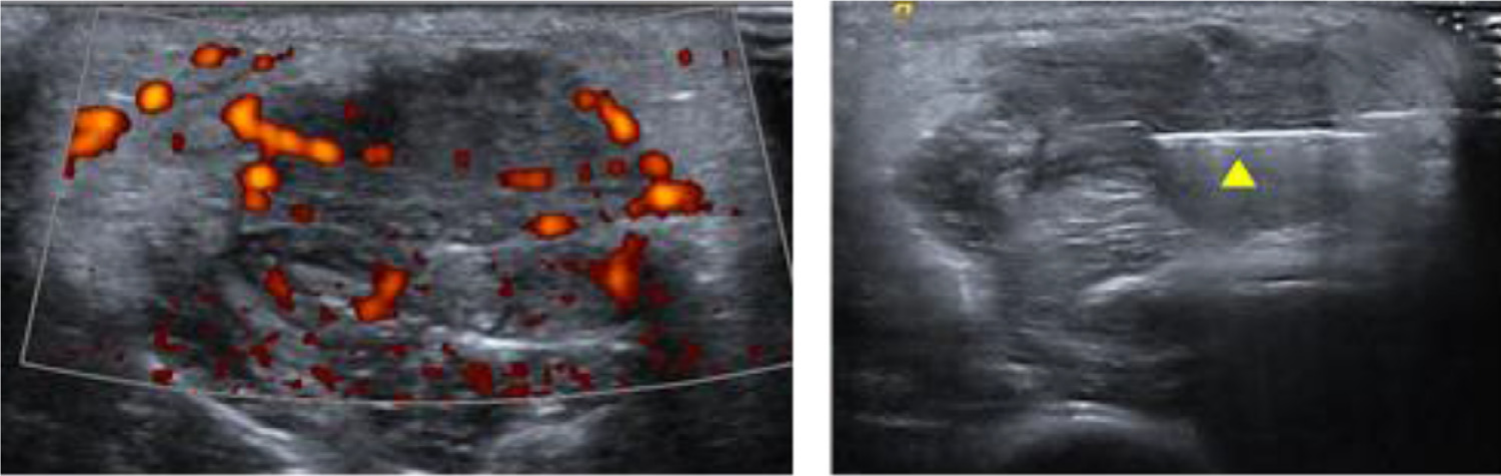
(A) Short axis color Doppler US showing solid hyper vascular soft tissue mass involving the deltoid muscle belly. (B) US-guided Temno needle (yellow arrowhead) biopsy of the mass.

**Fig. 4:**
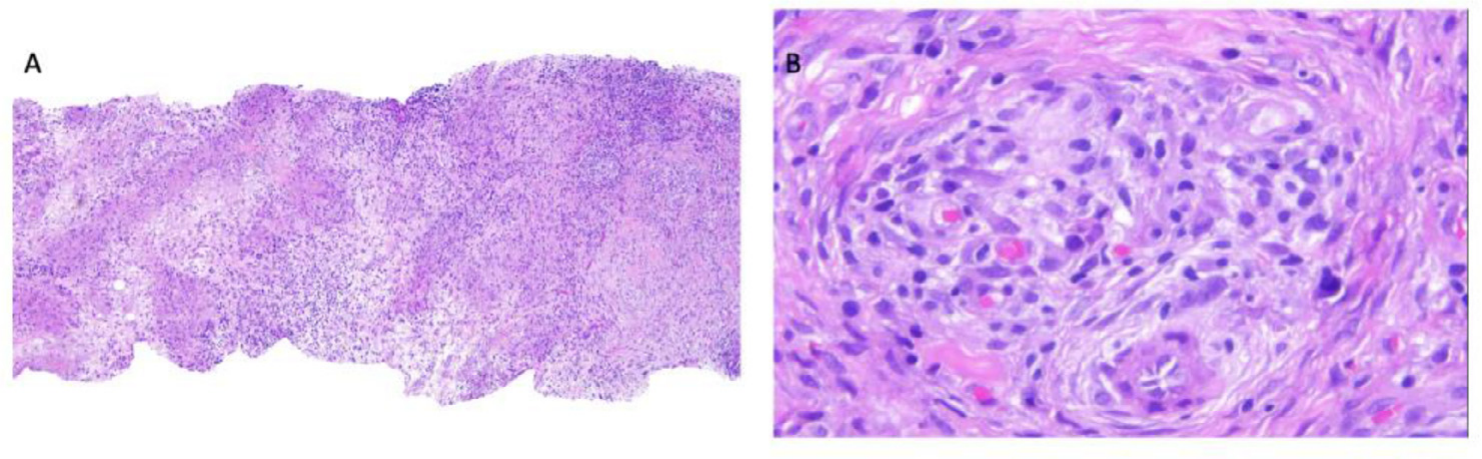
(A) Hematoxylin and eosin stain of a needle core of tissue showing multiple granulomas surrounded by granulation tissue. (B) Hematoxylin and eosin stain of the granulomas consisted of a well-delineated cluster of epithelioid histocytes and scattered lymphocytes. No evidence of neoplasia was demonstrated.

On physical exam, a nonmobile firm mass was palpable along the superolateral margin of the right deltoid muscle, without overlying skin changes. Neurovascular and motor testing were negative for weakness and sensory deficits. Shoulder radiographs revealed a soft tissue mass in the region of the lateral deltoid, without calcification or underlying osseous involvement (Fig. 1). Subsequent MRI demonstrated a soft tissue mass measuring 5.2 × 6.4 × 7.2 cm (anteroposterior × transverse × craniocaudal dimensions), with irregular borders involving the lateral belly of the deltoid and surrounding subcutaneous fat (Fig. 2). An ultrasound guided biopsy was performed (Fig. 3) and pathological analysis demonstrated non-necrotizing granulomatous inflammation without evidence of neoplasia (Figs. 4A and B). Given the patient's symptoms and the prominence of the mass, surgical excision was offered. Four weeks after the initial presentation, physical exam demonstrated a significant decrease in the size of the mass. Two months afterwards the mass was no longer palpable and the patient had no discomfort.

## Discussion

We present a case of a 66-year-old woman who developed a localized granulomatous reaction that presented as a mass following a COVID-19 Moderna Booster vaccine [12], [13]. The lesion resolved spontaneously without medical intervention. The differential diagnosis of the mass included other inflammatory etiologies and neoplasms, such as soft tissue sarcoma, shoulder injury related to vaccine administration (SIRVA) or nodular fasciitis. This patient reported a history of RA, which is linked to the development of subcutaneous nodules in many patients [2]. Our patient did not exhibit the development or worsening of joint pain or morning joint stiffening and has been effectively treated with standard of care DMARD (Methotrexate) for several years [2]. The development of rheumatoid nodules usually occurs with severe, uncontrolled rheumatoid factor seropositive rheumatoid arthritis [3]. Furthermore, RA nodules tend to have well-encapsulated features on MRI and are localized to bony prominences or juxta-articular surfaces [2,4]. In our patient, the mass spontaneously decreased in size and on histology did not exhibit the diagnostic palisading arrangement of histiocytes characteristic of rheumatoid nodules. SIRVA is an inflammatory process that results when a vaccine is injected into the shoulder capsule as opposed to the deltoid muscle [14]. On clinical exam, patients with SIRVA exhibit concerns of pain and functional limitations that occurred shortly after vaccine administration. Our case is different as the patient's main concern was not pain, but the increasing size of the soft tissue lesion. Furthermore, on MR imaging we failed to identify inflammatory changes within the sub-acromial or sub-deltoid bursae which are typically seen in cases of SIRVA [14]. The final differential diagnosis considered was Nodular Fasciitis which is a common fibrous lesion with a rapidly enlarging course and reported spontaneous remission. Nodular fasciitis can be painless and occur in the upper extremity of middle-aged individuals. However, on histology there are haphazardly arranged spindled cells with irregular bundles and no granulomatous formation. As demonstrated in Fig. 4A there are numerous granulomas, with numerous histiocytes.

Several case reports have documented the development of granulomatous inflammation following vaccine administration. One study reported postvaccination granulomas in children following the pertussis immunization. This same group identified that the ingredient responsible was an aluminum salt adjuvant as 77% of children who developed granulomatous inflammation also developed a contact allergy to aluminum [5]. We hypothesize the etiology in this specific case is a hypersensitivity response to a component(s) of the booster. Delayed hypersensitivity reactions occur when a circulating antigen is presented to CD4 T-cells via antigen presenting cells, commonly referred to as Langerhans cells. Using cytokines, such as interferon gamma, interleukin 1 and 6, macrophages and neutrophils are recruited to the site to induce an inflammatory response. If the antigen is neither consumed nor eliminated, the inflammatory process can persist and lead to the formation of granulomas. Granulomas are caused by an attempt from macrophages to form a barrier between tissue and an indigestible antigen. The Moderna COVID-19 vaccine does not contain aluminum, but does include other antigens, including polyethylene glycol (PEG) and tromethamine [5,11] that can be associated with severe hypersensitivity reactions [6,7]. Studies using polyethylene glycol-based sealants in a rabbit aorta model demonstrated an intense granulomatous reaction involving lymphocytes, plasma cells, and multinucleated giant cells. Additionally, a separate case report regarding a PEG-based cutaneous filler REMAKE documented a similar granulomatous reaction with repeated use [8]. Tromethamine, on the other hand, has been linked to severe anaphylactic reaction to the intravenous medication Toradol, but no granulomatous reaction has been recorded to date [6]. Nevertheless, a delayed hypersensitivity reaction to either of the two antigens may produce a robust granulomatous response. Spontaneous remission of the granuloma can be partly explained by reactive oxygen species mediated degradation [9,10]. Removal of the one of the offending agents (PEG) is possible via macrophage activation and release of reactive oxygen species, which destabilize and destroy the antigen [7]. This reported ingredient of Modena's COVID-19 vaccine may be responsible for a granulomatous reaction with rapid onset that mimicked an aggressive soft tissue tumor and subsequent remission.

## Patient consent statement

We hereby confirm consent was granted for the publication of this case by the patient, as so long as all records are de-identified.
