# Erratum to “COVID-19 vaccine-associated granulomatous mass mimicking a sarcoma: A case report” [Radiology Case Reports 17 (2022) 2775-2778]

**DOI:** 10.1016/j.radcr.2022.09.081

**Published:** 2022-10-17

**Authors:** Daniel Quintero, Nikhil Patel, Griffin Harris, Anthony Maristany, Ali Alani, Andrew E Rosenberg, Sheila A Conway, Jean Jose

**Affiliations:** a University of Miami, Leonard Miller School of Medicine, Miami, FL, USA; b Department of Orthopedic Surgery, University of Miami, Leonard Miller School of Medicine, Miami, FL, USA; c Florida International University Herbert Wertheim College of Medicine, Miami, FL, USA; d Department of Pathology, University of Miami, Leonard Miller School of Medicine, Miami, FL, USA; e Department of Radiology, University of Miami, Leonard Miller School of Medicine, Miami, FL, USA

The publisher would like to inform you that during typesetting, a spelling error was made in the title of this article and as a result “granulomatous” was incorrectly spelled as “ganulomatous.”

The publisher apologizes for any inconvenience caused by this mistake.

